# Fully-coupled fluid-structure interaction simulation of the aortic and mitral valves in a realistic 3D left ventricle model

**DOI:** 10.1371/journal.pone.0184729

**Published:** 2017-09-08

**Authors:** Wenbin Mao, Andrés Caballero, Raymond McKay, Charles Primiano, Wei Sun

**Affiliations:** 1 Tissue Mechanics Laboratory, The Wallace H. Coulter Department of Biomedical Engineering, Georgia Institute of Technology and Emory University, Atlanta, GA, United States of America; 2 Cardiology Department, The Hartford Hospital, Hartford, Connecticut, United States of America; Worcester Polytechnic Institute, UNITED STATES

## Abstract

In this study, we present a fully-coupled fluid-structure interaction (FSI) framework that combines smoothed particle hydrodynamics (SPH) and nonlinear finite element (FE) method to investigate the coupled aortic and mitral valves structural response and the bulk intraventricular hemodynamics in a realistic left ventricle (LV) model during the entire cardiac cycle. The FSI model incorporates valve structures that consider native asymmetric leaflet geometries, anisotropic hyperelastic material models and human material properties. Comparison of FSI results with subject-specific echocardiography data demonstrates that the SPH-FE approach is able to quantitatively predict the opening and closing times of the valves, the mitral leaflet opening and closing angles, and the large-scale intraventricular flow phenomena with a reasonable agreement. Moreover, comparison of FSI results with a LV model without valves reveals substantial differences in the flow field. Peak systolic velocities obtained from the FSI model and the LV model without valves are 2.56 m/s and 1.16 m/s, respectively, compared to the Doppler echo data of 2.17 m/s. The proposed SPH-FE FSI framework represents a further step towards modeling patient-specific coupled LV-valve dynamics, and has the potential to improve our understanding of cardiovascular physiology and to support professionals in clinical decision-making.

## Introduction

The left ventricle (LV) is a key structure of the cardiovascular system and, when diseased, is associated with a large share of cardiovascular disease (CVD)-related deaths [1]. In addition to continuous advances in medical imaging modalities such as phase-contrast magnetic resonance (PCMR), 4D echocardiography, and echo particle image velocimetry (E-PIV), computational fluid dynamics (CFD) simulations have been actively pursued and hold promise in studying detailed cardiac function in healthy and diseased states [2]. Modeling accurate patient-specific LV-valve dynamics, however, is challenging, mainly because cardiac function involves complex coupled interactions of the blood flow, the cardiac wall and the heart valves, an area where fluid-structure interaction (FSI) modeling is needed.

The structure and kinematics of the hearts valves are expected to have a significant impact on the intraventricular flow field [3]. Seo et al. [4] found that the mitral valve (MV) promote the formation of a circulatory flow pattern in the LV, increasing the strength of the apical flow and enhancing washout of ventricular blood. Results by Dahl et al. [5] also suggested that a physiological representation of the left atrium (LA) can influence the dynamics of the mitral leaflets and the intraventricular flow patterns. Additionally, recent studies have suggested that patient-specific flow patterns in the LV outflow tract (LVOT) are greatly affected by the dynamically deforming LV during diastole [6]. These findings underscore the importance of comprehensive FSI models of the LV coupled with the mitral and aortic valves for reproducing many of the key features of cardiac function.

Most of the LV FSI studies to date, however, have modeled either the AV [7, 8] or the MV only[5], have been limited to idealized geometries [9], or have focused only on a short time frame of the cardiac cycle—mainly the diastolic phase [5, 10]. In general, previous LV FSI models have incorporated extensive simplifying assumptions regarding the geometry, tissue mechanical properties and boundary conditions, which can add uncertainties in the simulation results. Current FSI approaches employed to study LV dynamics are mainly based on conventional mesh-based methods such as the Arbitrary-Lagrangian-Eulerian (ALE) formulation [11] or the immersed boundary (IB) method [12]. The most significant challenge of mesh-based FSI studies is dealing with the coaptation between the leaflets during valve closure, where the flow domain is separated into two unconnected regions, an issue that has been circumvented but not yet solved [13]. Recently, there has been an increased interest in mesh-free methods, such as the smoothed particle hydrodynamics (SPH) method [14]. The fully Lagrangian nature of SPH offers several advantages, such as handling small material-to-void ratios, solving solid and fluid dynamic equations in the same algorithm [15], and modeling large deformations with domain fragmentation.

SPH has been successfully applied in different fields of fluid and solid mechanics over the past two decades [14]. More recently, some researches have used SPH in FSI problems and validated the SPH-finite element (FE) coupling approach by benchmark cases such as beam impact and elastic gate [16, 17]. To date, most of the mesh-free cardiovascular studies have mainly focused on the blood flow through vessels or idealized LV geometries [18–20]. Shahriari et al. [21] studied and validated the capability of SPH to simulate the blood flow in a rigid 2D LV model. The FSI capability of the SPH-FE approach has also been used to simulate blood flow in a transcatheter AV model [20]. Recently, Caballero et al. [22] extended the application of SPH to simulate the large-scale intraventricular hemodynamics in two realistic 3D LV models with simplified valve structures. The comparison between SPH results, a traditional CFD method, and *in vivo* PCMR and echocardiography data showed a good quantitative agreement.

In the present study, an integrated LV-MV-AV FSI model using SPH for the fluid domain coupled with a nonlinear FE formulation for the passive mechanics of the heart valves was developed. Although some model simplifications are made, this work is considered the first attempt to simulate the coupled 3D AV and MV nonlinear soft tissue dynamics and the intraventricular hemodynamics in a realistic left-side heart model throughout the whole cardiac cycle. The cardiac wall motion was prescribed based on full phase multi-slice computed tomography (MSCT) scans, while leaflet dynamics were simulated using a fully-coupled FSI method. The LV structure used in this study takes into account various degrees of sophistication, including detailed mitral annulus (MA) and proximal LA motion, imaged-based asymmetric leaflet geometries, anisotropic hyperelastic constitutive models, and human material properties. Leaflet kinematics, valves structural response, and large-scale intraventricular hemodynamics were studied in detail, and compared to subject-specific echocardiography measurements. Finally, in order to investigate the coupling effects of the valves in the ventricular hemodynamics, the LV flow obtained from the FSI model was compared with the one obtained with a LV model without valves [22].

## Materials and methods

### 3D model reconstruction

**Fig 1:**
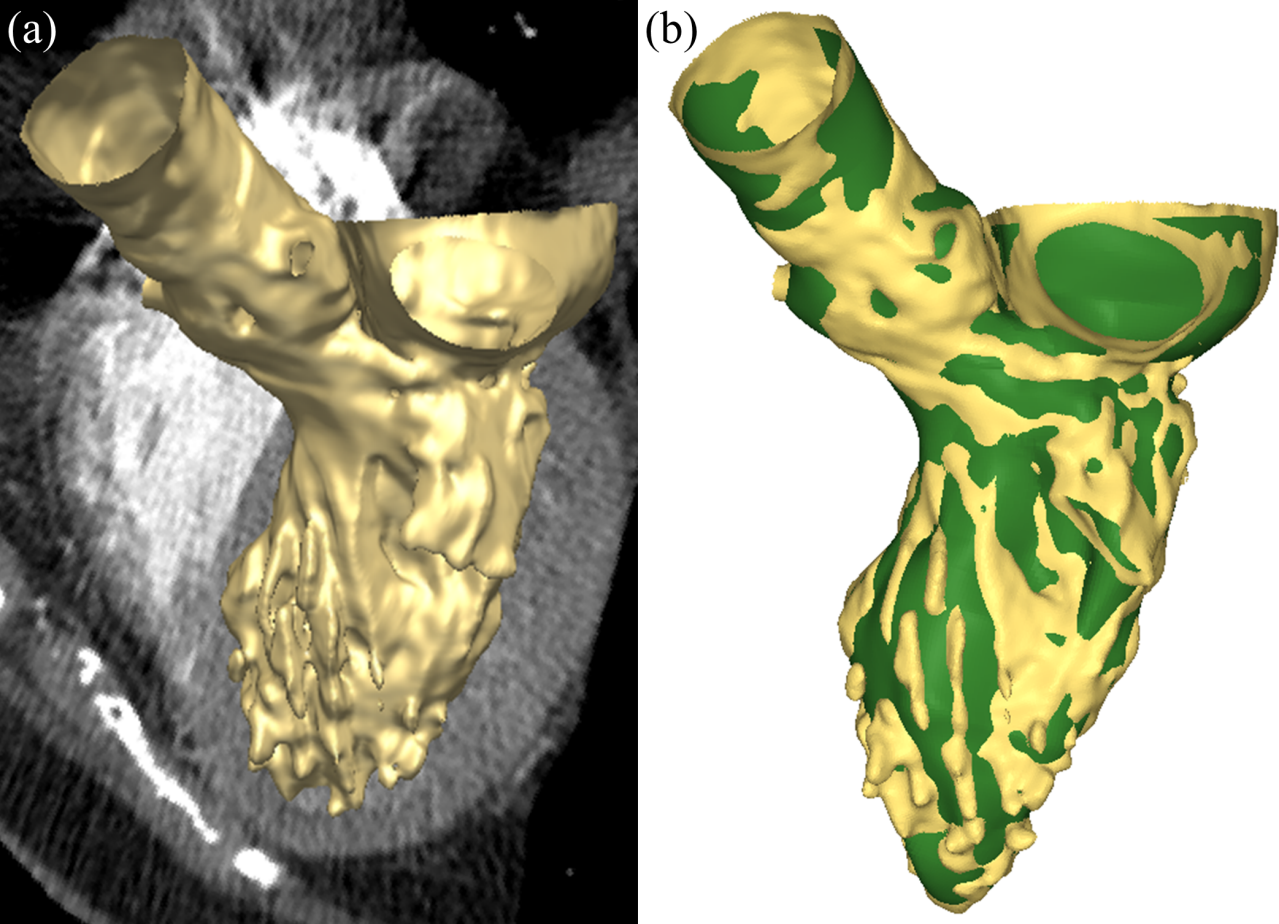
(a) Representative MSCT images of the LV during mid-systole overlapped with the 3D segmented geometry (yellow), (b) Segmented geometry overlapped with the reconstructed LV model (green).

The LV model was developed in a previous study from our lab [22]. Briefly, a 72-year-old female pre-operative transcatheter aortic valve replacement (TAVR) patient with normal LV function and no wall motion abnormalities was selected for this study. Institutional Review Broad approval to review de-identified clinical data was obtained. *In vivo* hemodynamic data, including transthoracic echocardiogram with complete 2D, M-Mode and Doppler examination was also obtained from the patient. The MSCT examination was performed on a GE LightSpeed 64-channel volume CT scanner. A total of 2540 slices of images with an in-plane resolution of 0.82 x 0.82 mm and a slice thickness of 0.625 mm were collected for the whole cardiac cycle (Fig 1). 10 phases obtained from one cardiac cycle were imported into Avizo (Thermo Fisher Scientific, MA) for 3D semi-automatic segmentation [23–25].

The AV geometry was reconstructed at the mid-systolic phase (stress-free state) from this patient, however, valve calcification was not incorporated in order to mimic a healthy state. In addition, since the complete MV geometry was not clearly detected from the MSCT scans for this particular patient due to artifacts in the valve location, the MV was adapted from a previous study from our group that validated healthy MV dynamics [23]. Special care was taken to ensure that the adapted MV resembled the same positioning of the subject-specific valve. The adapted MV geometry, reconstructed from the mid-diastolic phase (stress-free state), was scaled, translated and rotated until its boundary matched the MV boundary that was delineated from the subject-specific MSCT scans. The valve models considered various degrees of sophistication, including imaged-based asymmetric leaflet geometries, leaflet thickness and chordae tendineae with distinguishable chordal insertion points in the mitral leaflets [23].

**Fig 2:**
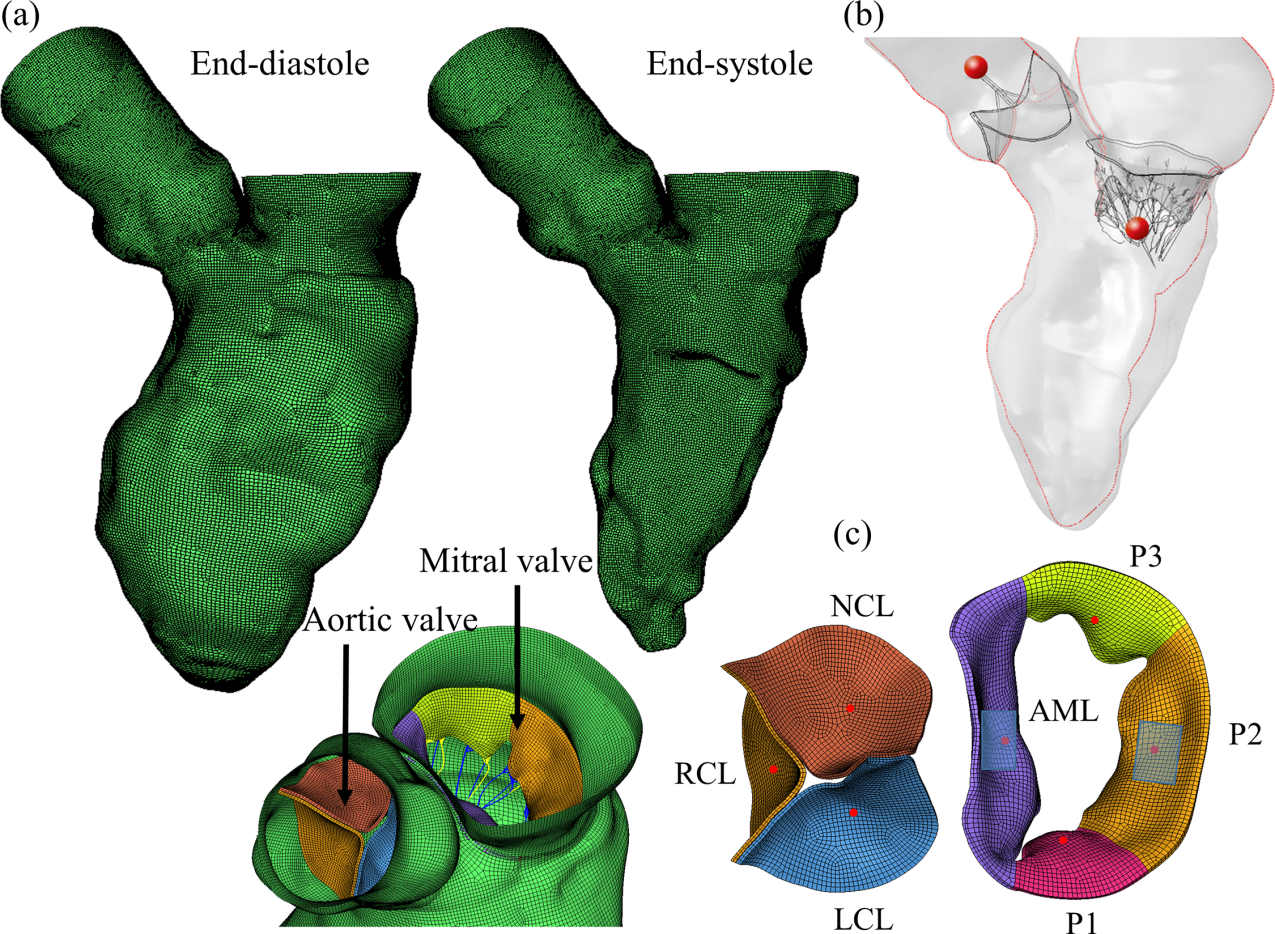
Schematic of LV-Valve model. **(a) LV model at different phases, (b) The anterior-posterior long axis plane is shown by the red line. The red spheres denote the probe locations for velocity measurements, (c) Initial stress-free state for AV and MV.** RCL: right coronary leaflet, LCL: left coronary leaflet, NCL: non-coronary leaflet, AML: anterior mitral leaflet. Posterior mitral leaflet (PML) is divided into lateral P1 scallop, central P2 scallop and medial P3 scallop.

The reconstructed 3D LV model with both valves can be seen in Fig 2A. The anterior-posterior long axis plane where the velocity measurements will be analyzed is indicated as a red line in Fig 2B. The red spheres denote the probe locations for velocity measurements through the valves. In Fig 2C, the blue rectangles represent the regions of interest for stress and strain calculations, while the red dots in the leaflets represent the location for leaflet kinematic measurements. In order to investigate the coupling effects of the valves in the LV flow dynamics, a LV model that did not incorporated the AV and MV, as developed in Caballero et al. [22], was used for comparison purposes. In the text, “LV-Valve” refers to the model with the valves, while “LV-NoValve” refers to the model without valves.

## Numerical modeling

### SPH modeling

In SPH, the continuum medium is discretized as a set of particles distributed over the computational domain without the need of a spatial mesh. The equations of motion and properties of the particles are determined from the continuum equations of fluid dynamics by a kernel interpolation technique [14]. This concept is interpreted numerically using a summation

$$A(r_{a})=\sum_{b}m_{b}\frac{A_{b}}{\rho_{b}}\;W(r_{a}-r_{b},\;h)$$ (1)

where *A*_b_ denotes any physical property at particle ‘b’ within the neighboring domain (limited by the influence length h of the kernel) of particle ‘a’ at position **r**_a_. Particle ‘b’ has mass *m*_b_, position **r**_b_, and density *ρ*_b_. In this study, a cubic spline kernel function *W* was adopted. Using this equation and its derivatives, the governing equations of fluid flow can be rewritten under the form of SPH formulation. The time derivative form of the conservation of mass gives

$$\frac{d\rho_{a}}{dt}=\sum_{b}m_{b}v_{ab}{∙\nabla }_{a}W_{ab}$$ (2)

Here ∇_a_*W*_ab_ is the gradient of the kernel function regarding the coordinates of given particle ‘a’ and ***v***_ab_ = ***v***_a_−***v***_b_ denotes the relative velocity vector between particles ‘a’ and ‘b’. Similarly, the conservation of momentum under the SPH scheme can be written as

$$\frac{dv_{a}}{dt}=-\sum_{b}m_{b}(\frac{P_{a}+P_{b}}{\rho_{a}\rho_{b}})\nabla_{a}W_{ab}+\sum_{b}m_{b}\frac{(\mu_{a}+\mu_{b})v_{ab}}{\rho_{a}\rho_{b}r_{ab}^{2}}r_{ab}∙\nabla_{a}W_{ab}$$ (3)

where *P* is pressure and *μ* is the dynamic viscosity of the fluid. More details on the implementation of the SPH formulation in Abaqus/Explicit (SIMULIA, Providence, RI) can be found in our previous publication [20].

In this study, we set a reference density of *ρ* = 1056 *kgm*^−3^ and a viscosity of *μ* = 0.0035 Pa ∙ s for blood properties. SPH particles were initially uniformly distributed in the domain with a spatial resolution of 0.8 mm. This led to approximately 497,400 one-node PC3D elements. The mesh sensitivity of SPH particles was previously checked [22], thus the same particle density was adopted here. Two cardiac cycles were conducted and the results from the second cycle were analyzed. It was found that the difference in velocity results between the first and second cycle was within 5% [22]. The stable time increment, determined by Abaqus/Explicit, was between 10^−6^ and 10^−7^ s for the LV-Valve model. This small time-step size ensured numerical stability and temporal accuracy.

### FE modeling

FE meshes were generated for the LV model using Hypermesh (Altair Engineering, MI). 3D solid elements (eight-node hexahedral C3D8R elements) were used to capture the thickness of the AV and MV, 3D stress/displacement truss elements (two-node linear T3D2 elements) were used to model the MV chordae, and shell elements (four-node reduced quadrilateral S4R elements) were used to represent the cardiac wall (i.e. LV, LA and LVOT). Two layers of elements were used across the leaflet thickness. The leaflet-chordae transition zone was modeled by creating fork-shaped truss elements at the chordal insertion points to mimic the native geometry and avoid stress concentration in the mitral leaflets [23].

For this study, two different constitutive models were used to model the mechanical response of valve tissues. First, the modified anisotropic hyperelastic Holzapfel–Gasser–Ogden material (MHGO) model [26] was adopted to characterize the mechanical behavior of human AV and MV leaflets. The strain energy function was expressed as

$$W=c_{1}\{\exp\left[c_{2}({\bar{I}}_{1}-3)\right]-1\}+\frac{k_{1}}{2k_{2}}\sum_{i=1}^{2}\left[exp\{k_{2}{\left[\kappa {\bar{I}}_{1}+(1-3\kappa ){\bar{I}}_{4i}-1\right]}^{2}\}-1\right]+\frac{1}{D}{\left(J-1\right)}^{2},\;i=1,2$$ (4)

where the strain invariant ${\bar{I}}_{1}$ is used to describe the matrix material and the strain invariant ${\bar{I}}_{4i}$ is used to describe the properties of the fiber families. *c*_1,2_ and *k*_1,2_ are the matrix and fiber parameters, respectively, *D* is a material constant to enforce incompressibility, and *J* is the determinant of the deformation gradient. In addition, *κ* is used to describe the distribution of fiber orientation. The anisotropic material model was implemented into Abaqus with a user sub-routine VMAT [27]. Local coordinate systems were defined for each leaflet to include local fiber orientation. The second material model used was the isotropic hyperelastic Ogden model [28], which was implemented to characterize the mechanical properties of human MV chordae tendineae. The Ogden strain energy function is given by

$$W=\sum_{i=1}^{N}\frac{2\mu_{i}}{a_{i}^{2}}({\bar{\lambda }}_{1}^{a_{i}}+{\bar{\lambda }}_{2}^{a_{i}}+{\bar{\lambda }}_{3}^{a_{i}}-3)$$ (5)

where *μ*_*i*_ and *a*_*i*_ are material constants, and ${\bar{\lambda }}_{i}$ are the modified principal stretches. In-house multiprotocol biaxial and uniaxial testing of healthy human AV and MV tissues (80-year-old female) was used to obtain the valves’ material properties [23]. Details of the determination of material parameters and FE implementation of these models have been described in our previous publications [29, 30]. The material parameters used for valve tissues are listed in Table 1.

**Table 1:** Valve tissue material parameters.

**MHGO model**	*C*_1_ (kPa)	*C*_2_	*k*_1_ (kPa)	*k*_2_	*θ*(°)	*κ*	*D* (kPa^−1^)
AV leaflets	1.738	11.368	2159.4	1158.9	4.59	0.2359	1.00e-5
Anterior MV leaflet	0.1245	13.665	11.007	84.848	13.09	0.0800	1.00e-5
Posterior MV leaflet	0.0502	15.004	3.021	144.48	25.51	0.0534	1.00e-5
**Ogden model**	*μ*_1_ (kPa)	*a*_1_	*μ*_2_ (kPa)	*a*_2_	*μ*_3_ (kPa)	*a*_3_	
Basal chordae	10256.1	16.579	10653.8	16.554	10671.3	16.554	
Strut chordae	24341.7	11.338	10331.9	11.167	14913.6	11.188	
Marginal chordae	12995.5	15.651	13082.9	15.683	12869.7	15.662	

### Boundary conditions

**Fig 3:**
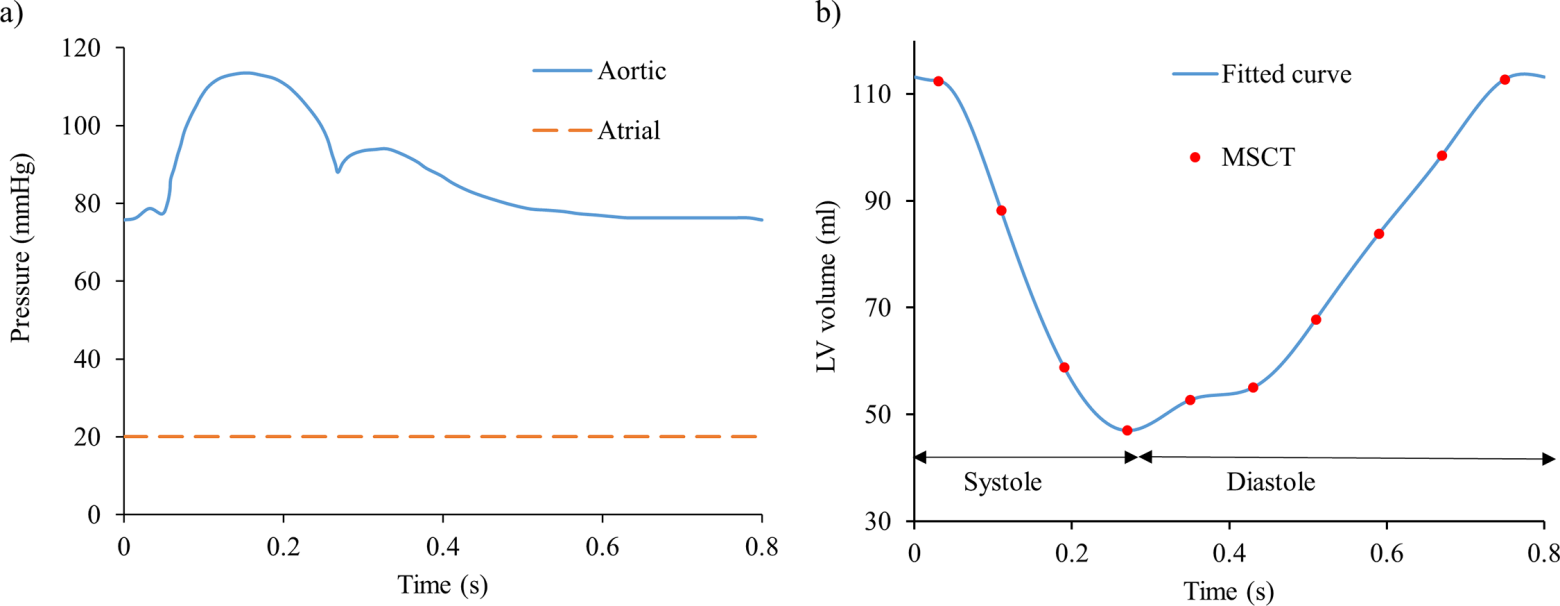
a) Aortic and atrial pressure boundary conditions, b) LV volume change waveform.

In order to accommodate the inflow and outflow of particles for two full cardiac cycles, the boundary location was extended upstream from the proximal LA and downstream from the LVOT by two straight tubes. Two plate pistons located at the extensions were used to apply the boundary conditions on the LV models. Numerically, the LV-Valve model employed a constant LA pressure of 20 mmHg at the inlet, while a physiological aortic pressure waveform, as seen in Fig 3A, was applied at the outlet [31, 32]. The shape of the aortic pressure waveform was carefully adjusted to be in phase with the subject-specific LV volume change waveform. Note that the atrial pressure slightly exceeds the normal physiological value in order to compensate for the long inlet extension used in the LV model. The LV-NoValve model, on the other hand, employed at the inlet the diastolic flow rate waveform derived from the LV volume change, with its value set to zero during systole [22]. For the outlet, the same aortic pressure waveform as in the LV-Valve model was applied. The patient’s heart rate was approximately 75 bpm, corresponding to a cardiac cycle of 0.8 s. Note that in our models, the cardiac cycle begins at early systole, resembling the isovolumetric contraction phase.

A detailed description of the prescribed cardiac wall motion procedure can be found in our previous publication [22]. As MA dimensions and shape vary dynamically during the cardiac cycle, the LV model developed in this study closely resembled the subject-specific MA and proximal LA dynamics by accurately tracking and interpolating the nodal displacements of these anatomical structures from the 10 MSCT scans. The aortic and mitral leaflet boundaries were prescribed the same motion as the cardiac wall in the aortic and mitral annuli to move accordingly. Additionally, chordal origins were not directly attached to the papillary muscle (PM) tips in the LV-Valve model (see Fig 2B), but were tracked and displaced between two spatial locations representing PM tips location at mid-diastole and mid-systole phases [23]. The coupling between SPH and FE was handled by the node-to-surface contact algorithm since both methods are based on the Lagrangian description. Briefly, the contact force at the interface was calculated by finding the best penalty force to prevent interface penetration and satisfy conservation of momentum. The combined effect of the smoothing kernel interpolation function near the boundary and the node-to-surface contact interaction approximates the no-slip and no-penetration boundary conditions, which was justified in our previous work [22].

## Results

### Global flow parameters

Fig 3B shows the time-varying LV volume change waveform, where the points indicate the 10 MSCT phases and the line represents the cubic spline interpolation. The end-diastolic (EDV) and end-systolic (ESV) volumes were 112 ml and 47 ml, respectively. The stroke volume (SV) and the ejection fraction (EF) were 65 ml and 58%, respectively, which closely matched the subject-specific data obtained from Doppler echo of 64 ml and 57%, respectively.

### Valve kinematics

**Fig 4:**
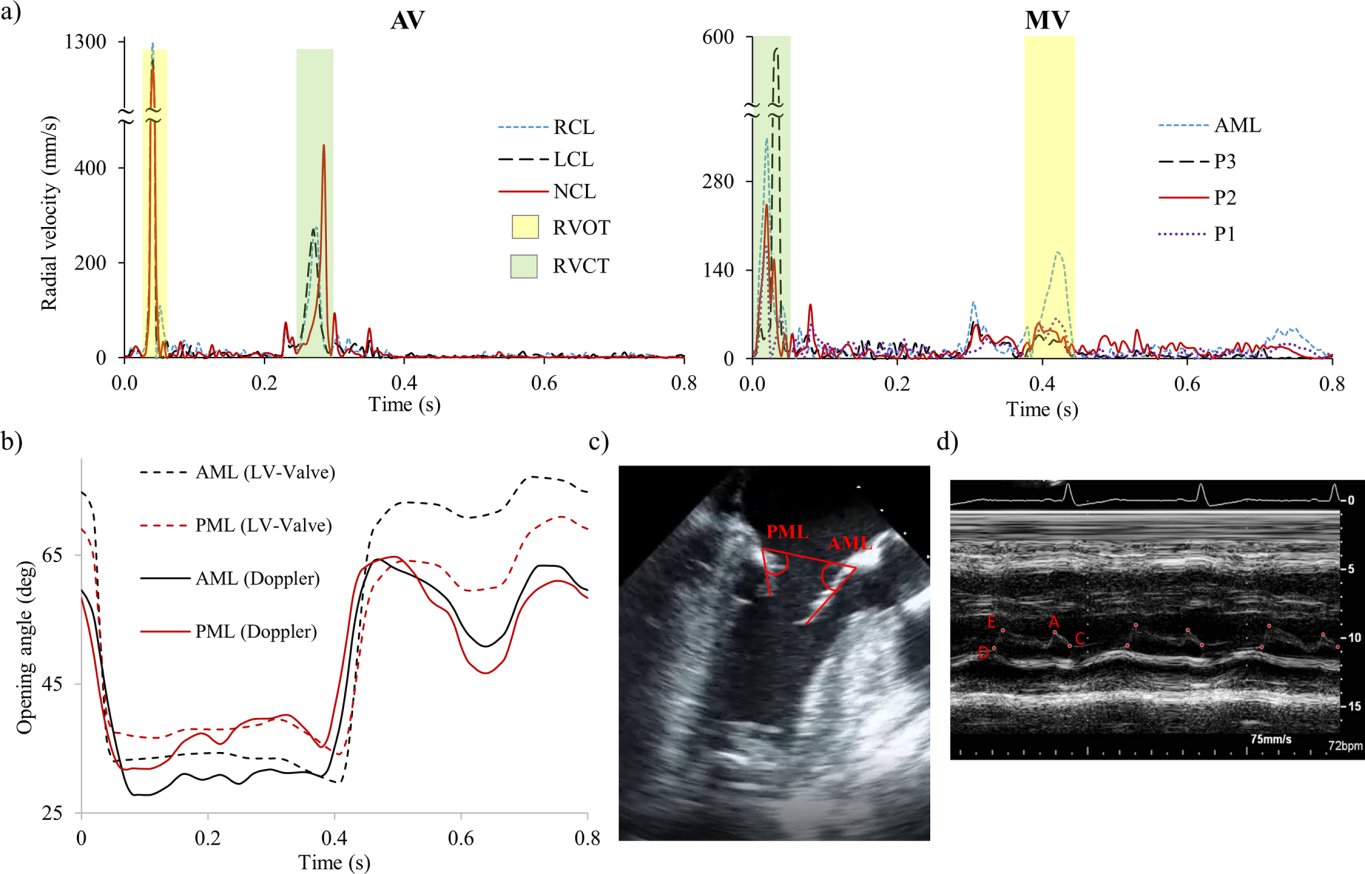
a) Time-dependent radial velocity of the belly (midpoint) of AV and MV leaflets. The measured RVOT and RVCT are highlighted in pale yellow and green, respectively, b) Opening angle of MV leaflets for LV-Valve model and echo data, c) schematic of angle measurements from echo video, d) M-mode echo recording at the MV.

Valve kinematics were analyzed in terms of AV and MV opening and closure dynamics during the entire cardiac cycle, and monitored through the time-dependent radial velocity of the midpoint (belly) of each leaflet (see red dots in Fig 2C). Fig 4A shows the computed rapid valve opening (RVOT) and closure (RVCT) times, defined as the duration tracts of the radial velocity curve with a high positive and high negative slope [33]. Additionally, ejection time (ET) was calculated as the time from the initial opening to complete closure of the valve. The results reported in Fig 4A are also summarized in Table 2.

**Table 2:** Comparison of ET, RVOT, and RVCT between LV-Valve model, subject-specific echo data and literature.

	AV		MV		
time (ms)	LV-Valve	Literature [34]	LV-Valve	Literature [35, 36]	M-mode echo
ET	270	329 ± 63	470	N/A	473
RVOT	30	57.5 ± 11.1	75	75–100	50
RVCT	50	39.5 ± 5	55	100–150	94

ET: ejection time; RVOT: rapid valve opening time; RVCT: rapid valve closing time.

In Fig 4B, the opening and closing angles of the MV leaflets (dotted lines) are plotted against the angles measured from echo (solid lines) along the apical long axis view (Fig 4C). During early-diastole, the onset of rapid valve opening occurs after the LV pressure falls below the LA pressure. During this phase, the simulated leaflets followed a similar opening timing pattern as the echo measurements. During mid- to end-diastole, which represent some partial leaflet closing after the E-wave followed by a second opening due to atrial contraction (A-wave), the simulated leaflets followed a similar pattern as in the echo data, but with higher opening angles. Note that for both numerical and *in vivo* measurements, the AML maintained a higher opening angle than the PML during the diastolic phase.

Additionally, Fig 4D shows the subject-specific M-mode echo recording at the MV position. Letters D, E, A and C represent end-systole before MV opening, peak of early opening, peak of late opening at atrial contraction, and MV closure, respectively. Therefore, RVOT can be calculated as the time interval between D and E, and RVCT as the time interval between A and C. As seen in Table 2, simulation results were consistent with measurements from *in vivo* and *in vitro* experiments [34–36], and with the subject-specific M-mode echo data. The largest quantitative mismatch between the simulation and *in vivo* data was for the MV RVCT.

### Structural stress and strain distributions

**Fig 5:**
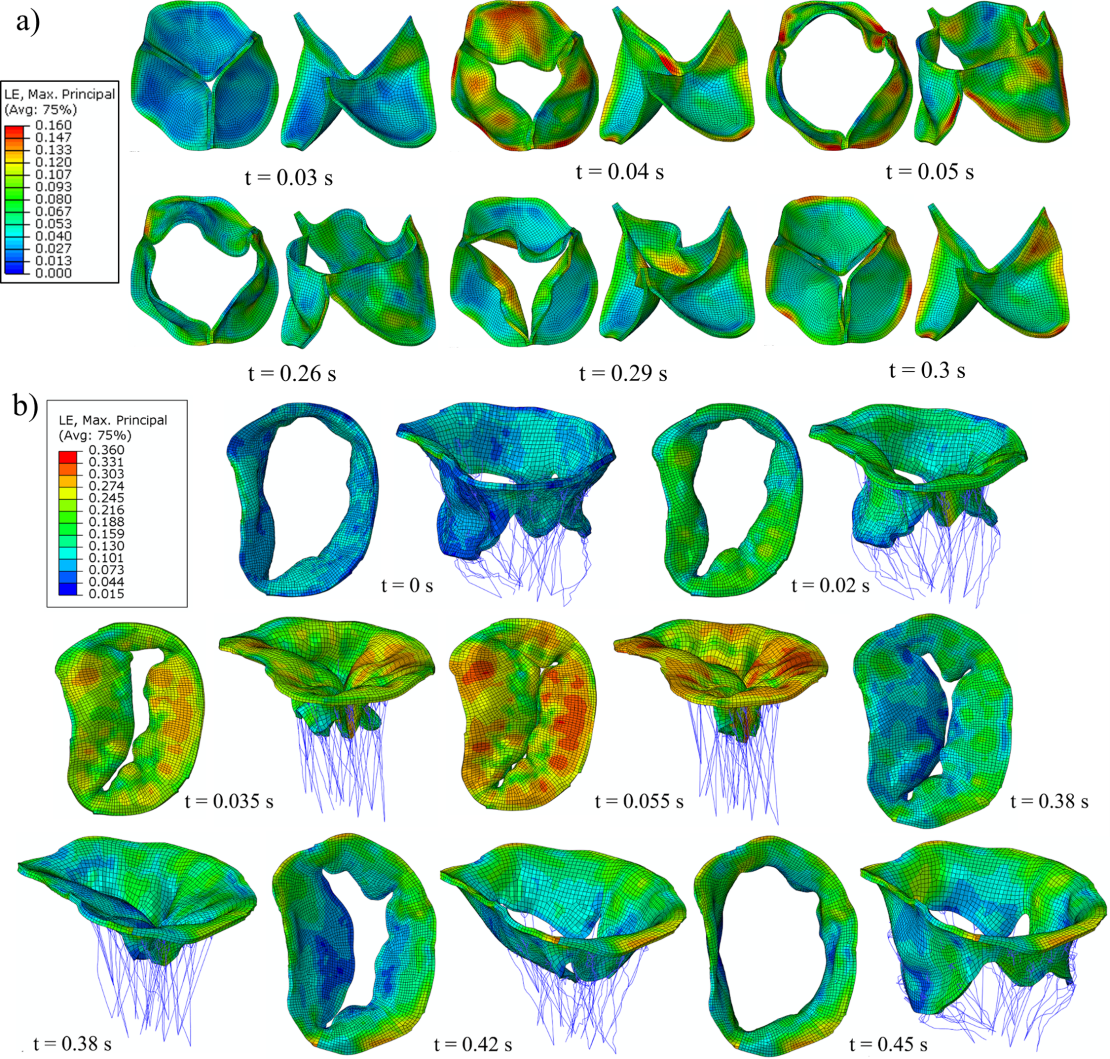
Opening and closing of a) AV and b) MV leaflets colored by the maximum principal strain. Strain in chordae tendineae not plotted.

Maximum principal logarithmic strain (LE) contours of the aortic and mitral leaflets at representative time points of the opening and closing phases are shown in Fig 5. For the AV, the peak strain during the ejection phase was observed along the leaflet attachment line, with a peak value around 16% (Fig 5A). During the valve closing phase, large strains were concentrated in the middle region of the leaflet attachment line, followed by the commissure regions. Some high strain areas were also observed in the ventricular layer near the central region, extending upwards towards the leaflet free edge during the closing and opening phases.

For the MV, Fig 5B shows that during the closing phase a heterogeneous strain distribution was found in the mitral leaflets, with high values located in the belly to annular P2 region, in the AML close to the right and left fibrous trigone annular regions, and at the insertion points of the chordae tendineae. During the opening phase, much lower strain values were obtained, with the maximum strain occurring along the MA. In general, significant heterogeneity was observed in the strain distribution for both valves due to tissue anisotropy and geometric asymmetry.

**Fig 6:**
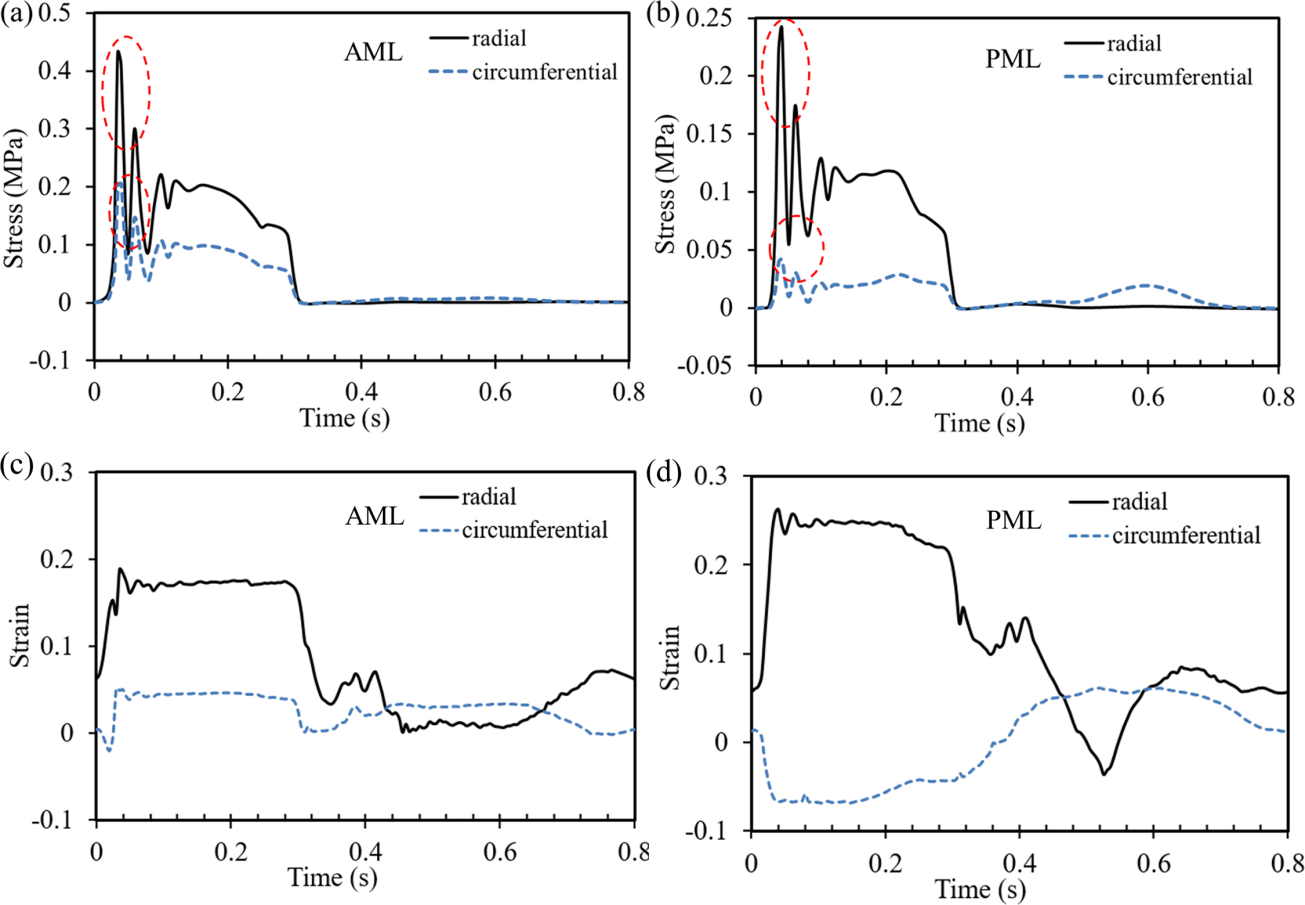
**Averaged circumferential and radial stresses of (a) AML and (b) PML, and circumferential and radial strains of (c) AML and (d) PML, within the region of interest, as marked in Fig 2C.** Note the spike values marked by the circles are due to numerical artifacts.

In addition, Fig 6 shows the radial and circumferential averaged (over the measuring region) stresses and strains for AML and PML belly regions, as marked in the rectangles of Fig 2C. From Fig 6A and 6B it can be seen that, in general, higher radial and circumferential stresses were observed in the AML than in the PML. Note that the spike values enclosed by the red circles during MV closure are due to numerical artifacts, which will be discussed later. In the diastolic phase, the stress values for both leaflets were negligible when the MV was open, with the PML circumferential stress being slightly higher at some time intervals.

### LV hemodynamics

**Fig 7:**
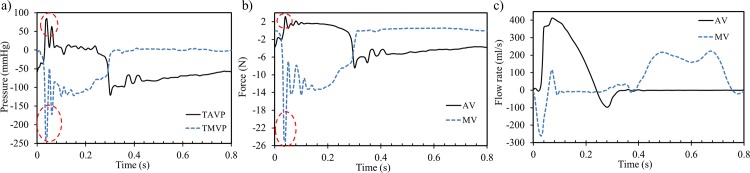
**a) Transvalvular pressure drop, b) hydrodynamic forces acting on the valves, and c) flow rate through the valves**. Note the spike values marked by the circles are due to numerical artifacts.

Table 3 compares global hemodynamic parameters calculated from the LV-Valve model and obtained from the subject-specific echo data. From the simulation, effective orifice area (EOA) was calculated for the AV (EOA_AV_) [37] according to the equation ${EOA}_{AV}(cm^{2})=\frac{MSF}{51.6\sqrt{∆P}}$, where *MSF* is the root mean square systolic flow rate (ml/s), and Δ*P* is the mean systolic pressure gradient (mmHg). Likewise, the EOA for the MV [38] was derived as ${EOA}_{MV}(cm^{2})=\frac{MDF}{31\sqrt{∆P}}$, where *MDF* is the root mean square diastolic flow, and Δ*P* is the mean diastolic pressure gradient. The competency of the valves was quantified via the regurgitant volume (RV) and the regurgitation fraction, *RF* = *RV*/*SV*, where RV is the sum of the closing volume and leakage volume. In general, the comparison between the numerical results and the *in vivo* measurements showed a reasonable agreement. However, it is noted that the difference between these values is significant during the diastolic phase. This is mainly due to the absence of calcifications at the MA in our adapted MV model compared to clinical observations. Additionally, Fig 7A shows the transvalvular pressure drop waveforms, measured one diameter upstream and three diameters downstream from the valve annuli. The shape and values of the pressure waveforms agree with well-known physiological measurements, except for the spike values enclosed by the circles during the early systolic phase, which will be discussed later.

**Table 3:** Comparison of hemodynamic parameters calculated from the LV-Valve model and the subject-specific echo data.

Parameters	LV-Valve	Echo Doppler
MSPD (mmHg)	11.1	10.1
PSPD (mmHg)	23.3	18.2
MDPD (mmHg)	2.0	3.6
PDPD (mmHg)	5.2	8.2
E/A	1.02	0.88
EOA_AV_ (cm^2^)	1.6	1.3
EOA_MV_ (cm^2^)	3.8	2.8
RV_AV_ (ml)	4.8	N/A
RF_AV_ (%)	7.4	N/A
RV_MV_ (ml)	9.8	N/A
RF_MV_ (%)	15	N/A

MSPD: Mean systolic pressure drop of AV; PSPD: peak systolic pressure drop of AV; MDPD: mean diastolic pressure drop of MV; PDPD: peak diastolic pressure drop of MV; E/A: ratio of E wave to A wave; EOA: effective orifice area; RV: regurgitant volume; RF: regurgitant fraction.

### Hydrodynamic forces

Fig 7B shows the axial hydrodynamic forces acting on the AV and MV, calculated by vector addition of the axial contact forces exerted on the leaflets surface due to the blood flow. Positive values represent the opening force, while negative values correspond to the closing force. During systole, peak values were 2.17 N and -14.6 N for the AV and MV, respectively. In diastole, peak forces reached up to -8.28 N and 0.54 N for the AV and MV, respectively. The MV opening force was found to be much lower than the value of the AV, which is due to the smaller mitral transvalvular pressure drop. During the closing phase, the hydrodynamic force on the MV was much higher than that on the AV.

Table 4 summarizes the average chordae tendineae tension at peak systolic pressure. These forces were found to be well within the range of *in vitro* experimental results [39]. Additionally, PM forces for the LV-Valve model were determined as the sum of the reaction forces on the chordal origin nodes that represent the tips of the PMs. At the systolic peak, the forces for the anterolateral and posteromedial PMs were 5.18 N and 4.7 N, respectively. These values agree with to the ones measured *in vitro* by Jensen et al. [40] and simulated by Wang et al. [23].

**Table 4:** Chordae tendineae peak systolic tension.

Chordae force (N)	LV-Valve	Jimenez et al. [39]
Marginal	0.221	0.23 ± 0.06
Strut	0.607	0.65 ± 0.13
Basal	0.235	0.22 ± 0.07

### Flow velocity

**Fig 8:**
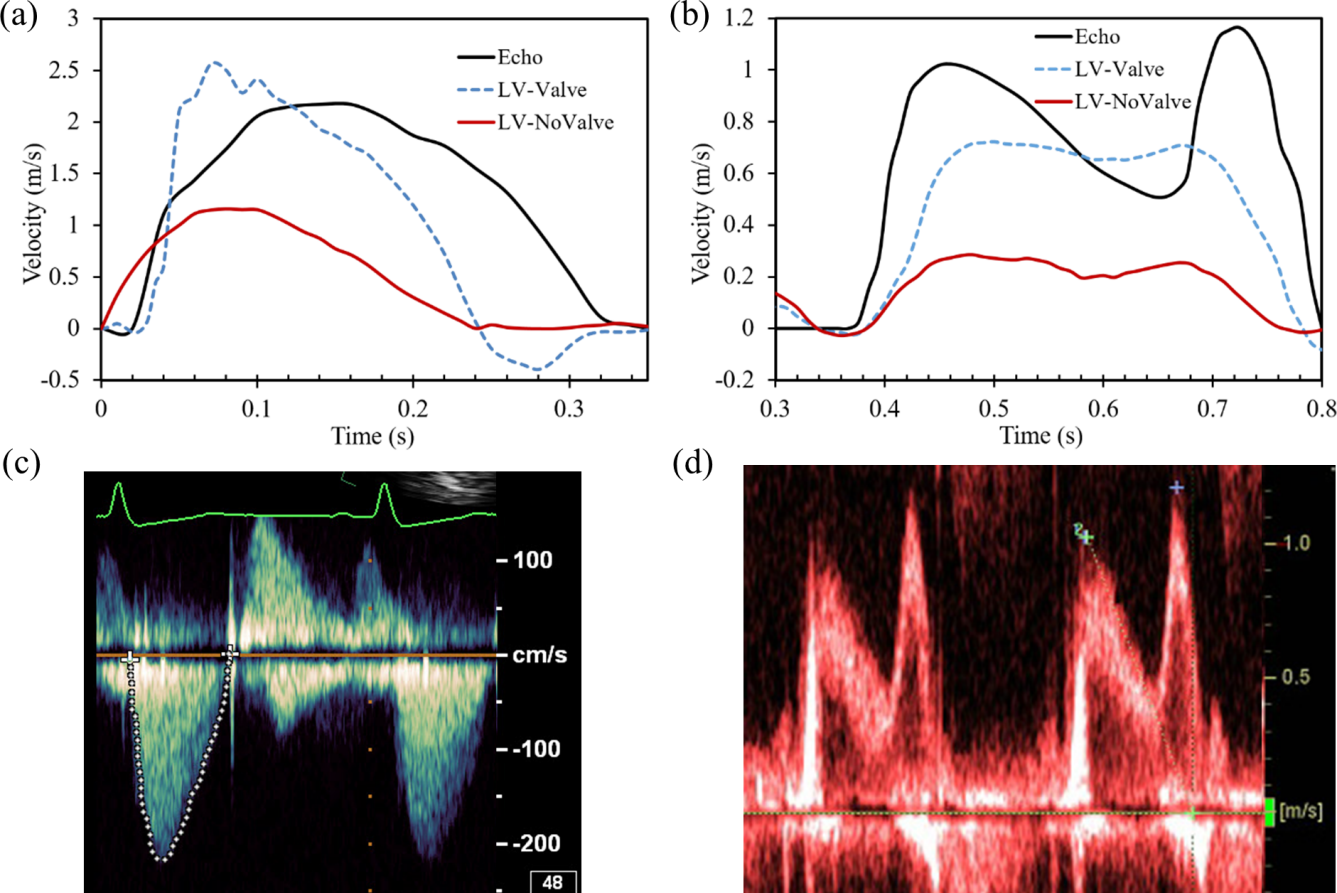
Flow velocity through the (a) AV and (b) MV. Doppler echo velocity recordings at c) AV and d) MV.

Fig 7C shows the calculated flow rate through the valves during the whole cardiac cycle. The retrograde flow can clearly be seen during valve closing. By integrating the negative flow rate in time, the RV of the AV and MV was calculated as 4.8 ml and 9.8 ml, respectively, representing a healthy valvular state. Note that RV is an important clinical hydrodynamic metric that can only be calculated from a fully-coupled FSI approach, which several previously published models [41, 42] were not able to quantify. In Fig 8A, the maximum flow velocity downstream of the AV (see Fig 2B) was monitored throughout systole and compared to the subject-specific Doppler recordings (Fig 8C). A quantitative comparison indicates that the AV velocity reached a similar peak value in both the LV-Valve model and the Doppler measurement. Furthermore, when compared to the LV-NoValve model, a much lower peak velocity of 1.16 m/s was observed. During the AV closing phase, a retrograde velocity is clearly seen for the LV-Valve model. In the LV-NoValve model, however, the retrograde velocity cannot be calculated. It is also important to be aware of the considerable limitations associated with Doppler velocity measurements. Since continuous wave (CW) Doppler examination cannot pinpoint where along the ultrasound beam the measured velocity comes from [43], retrograde flow measurements are challenging to obtain.

Similarly, the comparison of the flow velocity through the MV is shown in Fig 8B. The flow velocity was calculated at the midpoint between the tips of the opened MV leaflets (see Fig 2B), which corresponds to the probe location usually used in pulsed-wave (PW) Doppler measurements. It is noted that although the LV-Valve model had a similar MV waveform pattern as the echo data (Fig 8D), the peak value was not accurately predicted (0.74 m/s vs. 1.17 m/s). Moreover, it is observed that for the LV-NoValve model the velocity is much lower (0.28 m/s) than the echo data.

### Large-scale intraventricular flow patterns

**Fig 9:**
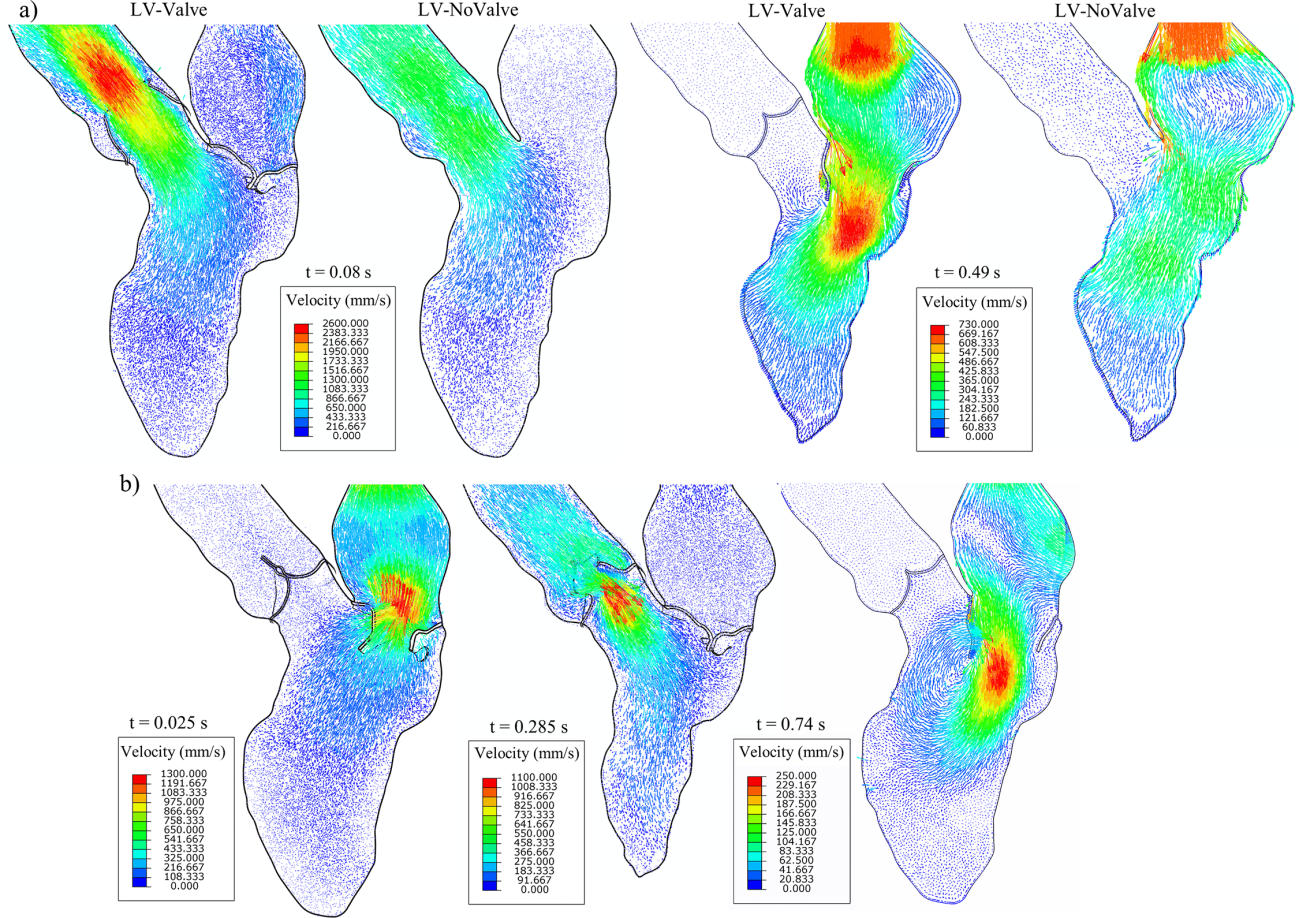
a) Velocity vector fields in the anterior-posterior plane for the LV-Valve and LV-NoValve models at peak systole and E wave, and b) for the LV-Valve model during MV closing, AV closing and late diastole.

Flow velocity vector fields in the anterior-posterior long axis plane (see Fig 2B) for the LV-Valve and LV-NoValve models at two different time points are shown in Fig 9A. At peak systole (~80 ms), a constricted and symmetric central jet was observed immediately downstream of the AV in the LV-Valve model, whereas a relatively uniform velocity profile from the LVOT to the ascending aorta was observed in the LV-NoValve model. As expected, without the constriction of the AV, the central jet velocity in the LV-NoValve model is much lower. Similarly, during the E-wave (~490 ms), the flow jet through the MV cannot be observed in the LV-NoValve model. Additionally, a vortex adjacent to the AML that is developed in the LV-Valve model is not present in the LV-NoValve model.

Finally, flow velocity vector fields from the LV-Valve model at additional time points are shown in Fig 9B. During MV (~25 ms) and AV (~285 ms) closing, a large retrograde flow through the valve is observed, while the opposite valve remained fully closed. A large asymmetric vortex that has been shown to initiate MV closure and pre-ejection filling of the LVOT [44] can be seen at late diastole (~740 ms).

## Discussion

Proper LV-valve dynamics requires a balanced interplay between the LV, MV, AV and the blood flow. Thus, blood flow-leaflet interaction, leaflet coaptation, and flow dynamics into, outward and within the LV are all critical parameters to investigate. From the LV-valve FSI models that have been developed, the earliest work is credited to McQueen and Peskin, that simulated MV dynamics in a contractile LV model [12, 45–47]. More recently, Chandran and Kim [48] studied MV dynamics during diastole using a simplified LV model. Using a fully-coupled FSI approach that included myocardial active contraction, Gao et al. [49] investigated LV-MV dynamics from diastole to systole. Although these studies have greatly advance the field of cardiac modeling, some of the limitations of these FSI models were the idealized representation of the LV and the mitral apparatus, and the complete omission of the AV geometry. To the best of our knowledge, this is the first 3D integrated LV-MV-AV FSI model that includes detailed valves structures, LV wall deformation, global LV flow dynamics, and nonlinear hyperelastic constitutive modeling of valvular tissue.

### Structural analysis

Understanding the coupled interaction between the valves and the LV is important for the assessment of cardiac function in healthy and diseased states, as well as for the evaluation and design of clinical procedures and medical devices for valve repair and replacement. In the current study, we quantified the valves maximum principal strain, and the circumferential and radial stress and strain of the AML and PML throughout the entire cardiac cycle. In particular, it was found that the leaflets experienced a highly anisotropic mechanical response. Note that during systole, Fig 6C and 6D shows that the radial strain was higher in the PML than in the AML. This feature was previously observed by Lee et al. [50] and Watton et al. [51]. In the circumferential direction, however, the PML showed negative strain values during the entire aortic ejection phase. The PML compressive strain is likely caused by the strong mechanical coupling between the circumferential and radial directions at systole. Indeed, in vivo and in vitro studies [35, 52] have shown that the coupling of the stretches in two directions can generate compressive stretches in the circumferential direction of the PML at the beginning of MV closure, when there is a large stretch in the radial direction.

Additionally, MV strains shown in Fig 6C and 6D were qualitatively similar to previous *in vivo* and *in vitro* studies [52–55], with a rapid strain increase to the point of full coaptation, followed by a stable plateau in both radial and circumferential directions during systolic ejection. In Table 5, the strains obtained numerically at the fully loaded state are quantitatively compared to those measured *in vivo* and *in vitro* in ovine and porcine MVs. Our numerical results show a close agreement with the *in vivo* and experimental results.

**Table 5:** Comparison of the MV strain values between the current study and the literature.

Study	Model	Strain (%)
**AML**		
LV-Valve	FSI	Circumferential: 4.5 Radial: 17.2
Sacks et al. [53]	*In vivo*	Circumferential: 3.2 ± 0.4 Radial: 16 ± 3
Amini et al. [54]	*In vivo*	Circumferential: 8 Radial: 21
Sacks et al. [55]	*In vitro*	Circumferential: 11 ± 3 Radial: 30 ± 8
Jimenez et al. [56]	*In vitro*	Circumferential: 11 ± 5 Radial: 22 ± 7
**PML**		
LV-Valve	FSI	Circumferential: -6.8 Radial: 25.1
He et al. [35]	*In vitro*	Circumferential: 3 Radial: 23
Padala et al. [57]	*In vitro*	Circumferential: 10 ± 8 Radial: 29 ± 8

### Fluid analysis

A quantitative flow field comparison between the LV-Valve model and the LV-NoValve models is presented in Fig 9A. The presence of the AV resulted in a decreased orifice area of 2.24 *cm*^2^ at the tip of the leaflets during peak systole. Meanwhile, without the valve, the geometric orifice area at the annulus was about 4.55 *cm*^2^. The large-scale flow patterns were quite different as well. Fig 9A shows that for the LV-Valve model, the maximum velocity occurred just downstream of the AV, where the flow jet is highly concentrated but the flow velocity inside the sinus region is less than 0.1 m/s. However, in the LV-NoValve model, the maximum flow velocity occurred at the annulus with an unrealistically high flow velocity of 0.5 m/s in the sinus region. Similarly, the MV flow velocity was much higher in the LV-Valve model than that in the LV-NoValve model. Due to the strong flow jet and the closure of the AV, a vortex adjacent to the AML was developed in the LV-Valve model, which was not seen in the LV-NoValve model. This vortex is considered to act as a reservoir that stores kinetic energy as it redirects blood towards the lateral LV wall and initiates MV closure and pre-ejection filling of the LVOT in late-diastole [44]. It is also noted that since complete closure of the MV and AV was achieved in the FSI simulation, several interesting characteristics such as regurgitant flow have been observed (Fig 9B) and quantified (Table 3).

The spike values enclosed by the red circles in the leaflets stresses (Fig 6A and 6B), transvalvular pressures (Fig 7A) and hydrodynamic forces (Fig 7B) are thought to be caused by numerical artifacts in the FSI simulation. During early systole, there is a short time period when both valves are nearly closed, forming a nearly closed system. Due to the assumption of incompressible fluid, a small compression in volume for a closed system can cause a large change in pressure. In our FSI model, the motion of the cardiac wall was prescribed according to the MSCT data, thus, a small decrease in LV volume, which is very likely to occur during early systole, could lead to a large fluctuation in pressure. This fluctuation, however, damped out rapidly due to the viscous effect of the fluid. This numerical artifact could be resolved by modeling the interaction between the myocardium and the blood flow by considering the active contraction of the myocardium. This is the subject of a study that we are currently undertaking.

Flow velocity passing through the MV from our models was compared to the Doppler echo data in Fig 8B. Even with the inclusion of the valves, our simulated flow velocity is lower than that of the subject-specific data. Two possible reasons may cause this discrepancy: 1) the MV geometry used in the LV-Valve model is not subject-specific but was adapted from a previous study from our group [23]. Specifically, MA calcification was detected in the subject-specific Doppler examination (see Fig 4C) but was not included in our model in order to mimic a healthy state. MA calcification could potentially restrict normal leaflet motion and cause a smaller opening area during diastole. Indeed, as shown in Fig 4B and Table 3, relatively larger mitral leaflets opening angle and EOA, as well as lower MDPD and PDPD values were obtained from the FSI simulation compared to the *in vivo* measurements; 2) only proximal LA wall motion was included in our model due to the limited imaging window of the MSCT images. The incomplete LA dynamics could affect the mitral flow during atrial passive filling and contraction [5, 58]. Despite these limitations, our results are largely consistent with subject-specific *in vivo* measurements, clinical observations and *in vivo* and *in vitro* studies of healthy LV-MV-AV dynamics.

### Limitations

There are several limitations in this study, which should be considered when interpreting our results. One modeling simplification was the topology of the endocardium, which was assumed as a smooth surface without the trabeculae and a detailed structure of the PMs. The influence of these structures should be explore in a future study. Second, the no-slip boundary condition in Abaqus SPH is not fully constrained. This limited imposition is likely to affect the flow solution in the boundary layers region and limit the study of the small-scale flow features seen in the LV blood flow [22]. Lastly, due to the lack of myocardial active contraction, the spike values in the pressure, and leaflet forces and stresses during early-systole seem inevitable. Therefore, modeling the active and passive myocardium response together with the intraventricular blood flow would be the next step towards a more accurate FSI model of the left-side of the heart.

## Conclusions

In this study, the first 3D LV-MV-AV FSI model that includes detailed valve structures, LV wall deformation, anisotropic hyperelastic material models, human leaflet material properties and ventricular flow throughout the entire cardiac cycle was developed. A detailed quantitative comparison of simulation results with those of subject-specific *in vivo* measurements, clinical observations and *in vivo* and *in vitro* studies have been presented. Despite some modeling simplifications and limitations, FSI results were in reasonable quantitative agreement. This study demonstrated the ability of the SPH-FE FSI framework to simulate the healthy coupled valves structural response and the bulk intraventricular hemodynamics in a realistic LV model during the entire cardiac cycle. The development of a FSI framework which is capable of reproducing the coupled left-side heart dynamics would not only enhance our basic understanding of the functional morphology of these structures, but is also needed to obtain clinically relevant results and to better understand the implications of medical therapies and procedures such as MV repair or replacement.
